# Prenatal disorders and congenital Zika syndrome in squirrel monkeys

**DOI:** 10.1038/s41598-021-82028-3

**Published:** 2021-01-29

**Authors:** Aline Amaral Imbeloni, Bianca Nascimento de Alcantara, Leandro Nassar Coutinho, Sarah Raphaella Rocha de Azevedo Scalercio, Liliane Almeida Carneiro, Karol Guimarães Oliveira, Arnaldo Jorge Martins Filho, Darlene de Brito Simith Durans, Wellington Bandeira da Silva, Bruno Tardelli Diniz Nunes, Livia Medeiros Neves Casseb, Jannifer Oliveira Chiang, Carlos Alberto Marques de Carvalho, Mariana Borges Machado, Juarez Antônio Simões Quaresma, Daniele Barbosa de Almeida Medeiros, Pedro Fernando da Costa Vasconcelos

**Affiliations:** 1 National Primate Center, Evandro Chagas Institute, Rodovia BR-316, km-07, Ananindeua, Para 67030-000 Brazil; 2 Post-Graduate Program in Virology, Evandro Chagas Institute, Rodovia BR-316, km-07, Ananindeua, Para 67030-000 Brazil; 3 Rural Federal University of Amazonia, Trancredo Neves, Belem, Para 250166077-830 Brazil; 4 Department of Pathology, Evandro Chagas Institute, Rodovia BR-316, km-07, Ananindeua, Para 67030-000 Brazil; 5 Department of Arbovirology and Hemorrhagic Fever, Evandro Chagas Institute, Rodovia BR-316, km-07, Ananindeua, Para 67030-000 Brazil; 6 University Center of Para, Governador Jose Malcher Avenue, 485, Belem, Para 66035-065 Brazil; 7 University of Pará State, Tv. Perebebuí-Marco, 2623, Belém, Para State 66087-662 Brazil

**Keywords:** Diseases, Medical research

## Abstract

During the Zika virus (ZIKV) outbreak in Brazil (2015–2016), the clinical manifestations associated with its infection were complex and included miscarriage and congenital malformations, not previously described. In this study, we evaluated the prenatal conditions of pregnant female squirrel monkeys (*Saimiri collinsi*) infected during different gestational thirds (GTs) and assessed all clinical aspects, diagnostic imaging, viremia and the immune response. In our study, 75% of the infected animals in the 1st GT group had significant clinical manifestations, such as miscarriage and prolonged viremia associated with a late immune response. Consequently, their neonates showed fetal neuropathology, such as cerebral hemorrhage, lissencephaly or malformations of the brain grooves, ventriculomegaly, and craniofacial malformations. Thus, our study demonstrated the relevance of pregnant squirrel monkeys as a model for the study of ZIKV infection in neonates due to the broad clinical manifestations presented, including the typical congenital Zika syndrome manifestations described in humans.

## Introduction

Zika virus (ZIKV) belongs to the genus *Flavivirus* and family *Flaviviridae* and is mainly transmitted by *Aedes aegypti, Ae. albopictus* and *Ae. africanus* mosquitoes^1^. ZIKV is closely related to other flaviviruses that cause diseases that are of public health importance, such as dengue, Japanese encephalitis, tick-borne encephalitis, West Nile fever, and yellow fever^2^.

ZIKV was first isolated in Uganda in 1947, and since then, the virus was detected in Africa and later in Asia, causing outbreaks with dengue-like febrile symptoms in some cases^3^. In 2007, ZIKV spread throughout the Pacific Islands and reached South America and the Caribbean Islands, probably in 2013^4,5^. In May 2015, the ZIKV Asian genotype was detected in Brazil, where it quickly spread to other Latin American countries and was associated with severe neurological disorders, such as microcephaly and Guillain Barré syndrome (GBS)^5–7^.

Initially, ZIKV was known to cause mild clinical disease, with symptoms characterized by fever, maculopapular rash, arthralgia and conjunctival hyperemia^8,9^. However, especially after its introduction in Brazil, the ZIKV clinical range broadened to include severe malformations in fetuses, a condition named congenital Zika syndrome (CZS)^10^, and viral RNA was detected for a long time during the different stages of pregnancy, indicating viral persistence^11^.

Among the main neuroimaging findings related to CZS are craniofacial distortion with a microcephalic appearance associated with calcifications predominating in the cortico-subcortical junction, malformations of cortical development, ventriculomegaly and abnormalities in the corpus callosum^7^, lisencephaly^10,12^, growth restriction^10^, eye abnormalities^13^, arthrogryposis^14^ and early fetal loss and fetal death^10^.

To better understand the pathogenesis of ZIKV infection, specifically CZS, many murine, porcine and nonhuman primate (NHP) models have been tested^15–20^. The neotropical NHP of *Samiri sp*. has been used as a model for many infectious diseases, especially for viral encephalitis studies^21–23^. This genus has several advantages over Old World NHPs, which are usually used in reproductive research^24^; these advantages include the ease in handling the animals so that pregnant animals can be contained without the need for anesthesia due to their small size and short gestational period, with a well-defined reproductive season throughout the year^25,26^. Thus, we used pregnant squirrel monkeys to investigate the outcome of ZIKV experimental infection at different stages (thirds) of the gestational period. This is the first study from a series describing the prenatal clinical-laboratory aspects of pregnant squirrel monkeys after ZIKV infection and the neuroimaging findings in CZS fetuses.

## Results

### NHP females experimentally infected with ZIKV

**Figure 1:**
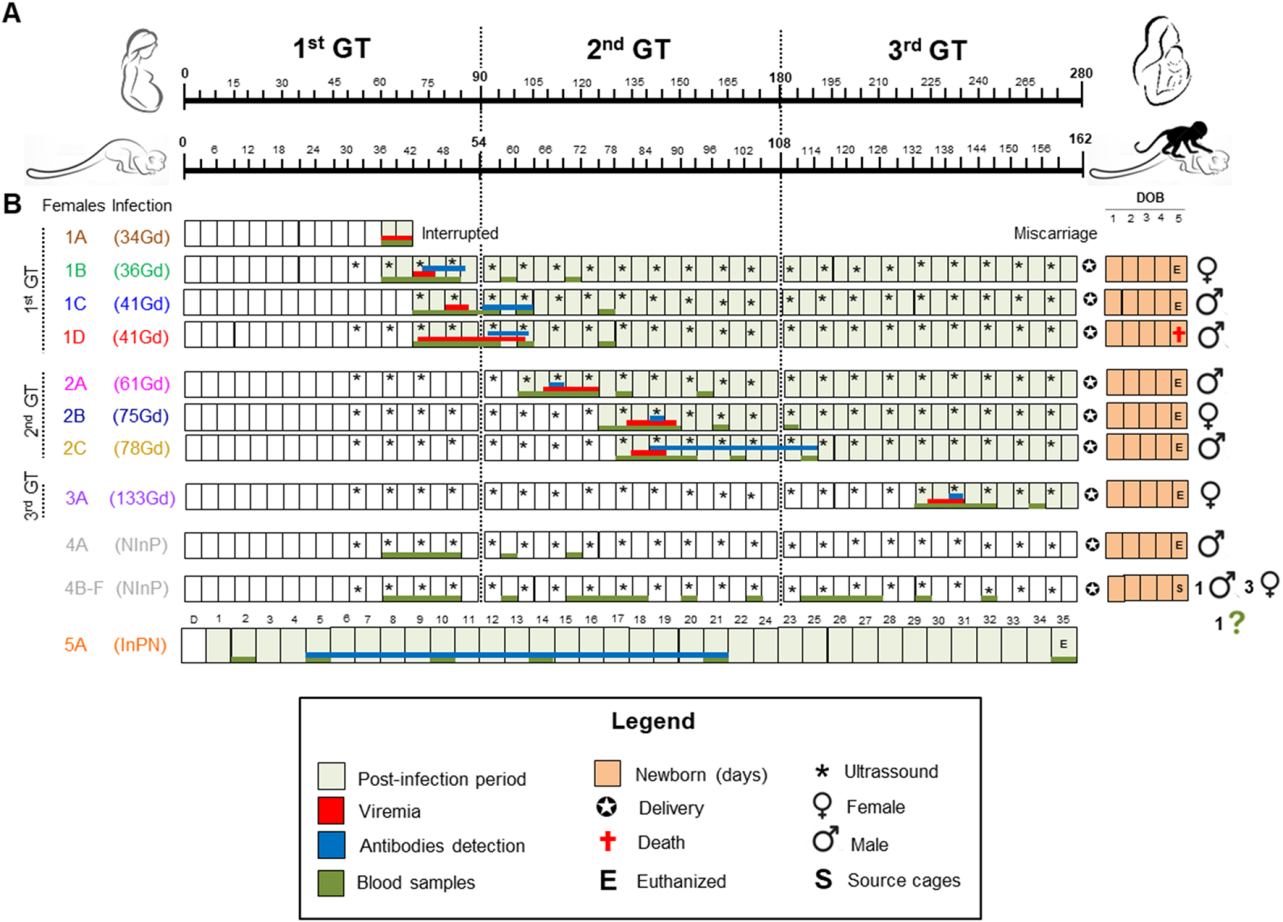
Experimental ZIKV infection in pregnant squirrel monkeys. Design of study, sex and outcome of neonates. (**A**) Comparison in days of the gestational periods between humans and squirrel monkeys. (**B**) Timeline of the five groups in the experiment: ZIKV-infected pregnant females in the 1st gestational third (GT), 2nd GT and 3rd GT, noninfected pregnant (NInP) group, and infected nonpregnant (InPN) group. The pregnant female squirrel monkeys were infected with ZIKV on the 34th, 36th, 41st, 61st, 75th, 78th and 133rd days (Gd) of the gestational period. The 1A animal (1st GT) miscarried; the other animals had normal gestations up to delivery.

Eight pregnant squirrel monkeys (*Saimiri collinsi*), by natural mating, were infected with the ZIKV Asian genotype (BeH815744) strain^5^ at a dose of 1.0 × 10^5^ PFU/ml in 0.5 ml of VERO cell-infected suspension, inoculated by the intradermic route (i.d.). The pregnancy was confirmed by ultrasound (US), and the gestational period was 162 ± 10 days, as previously described^26^. The animals were divided into three groups based on gestational third (GT) at the moment of infection: the 1st GT group, comprising four animals (1A, 1B, 1C, and 1D) infected during the 1st GT at 34, 36, 41, and 41 gestational days (Gd), respectively; the 2nd GT group, comprising three monkeys (2A, 2B, and 2C) infected at 61, 75 and 78 Gd, respectively; and the 3rd GT group, comprising one animal (3A) infected at 133 Gd. In addition, six noninfected pregnant females (NInP) were considered the control group, and one infected nonpregnant (InNP) female squirrel monkey was also included. The obstetric and clinical signs of all animals were monitored, as were hematological and biochemistry parameters (Fig. 1A,B). Prior to infection, all squirrel monkeys tested negative for flavivirus exposure in a hemagglutination inhibition test (HI).

All ZIKV-infected squirrel monkeys except for animal 1A, which had a miscarriage and was euthanized at 7 dpi, had normal deliveries. Furthermore, animal 4B from the NInP group ate part of its newborn at birth, making it impossible to identify its sex.

### Clinical, hematological and biochemical findings of ZIKV infection in squirrel monkeys

**Figure 2:**
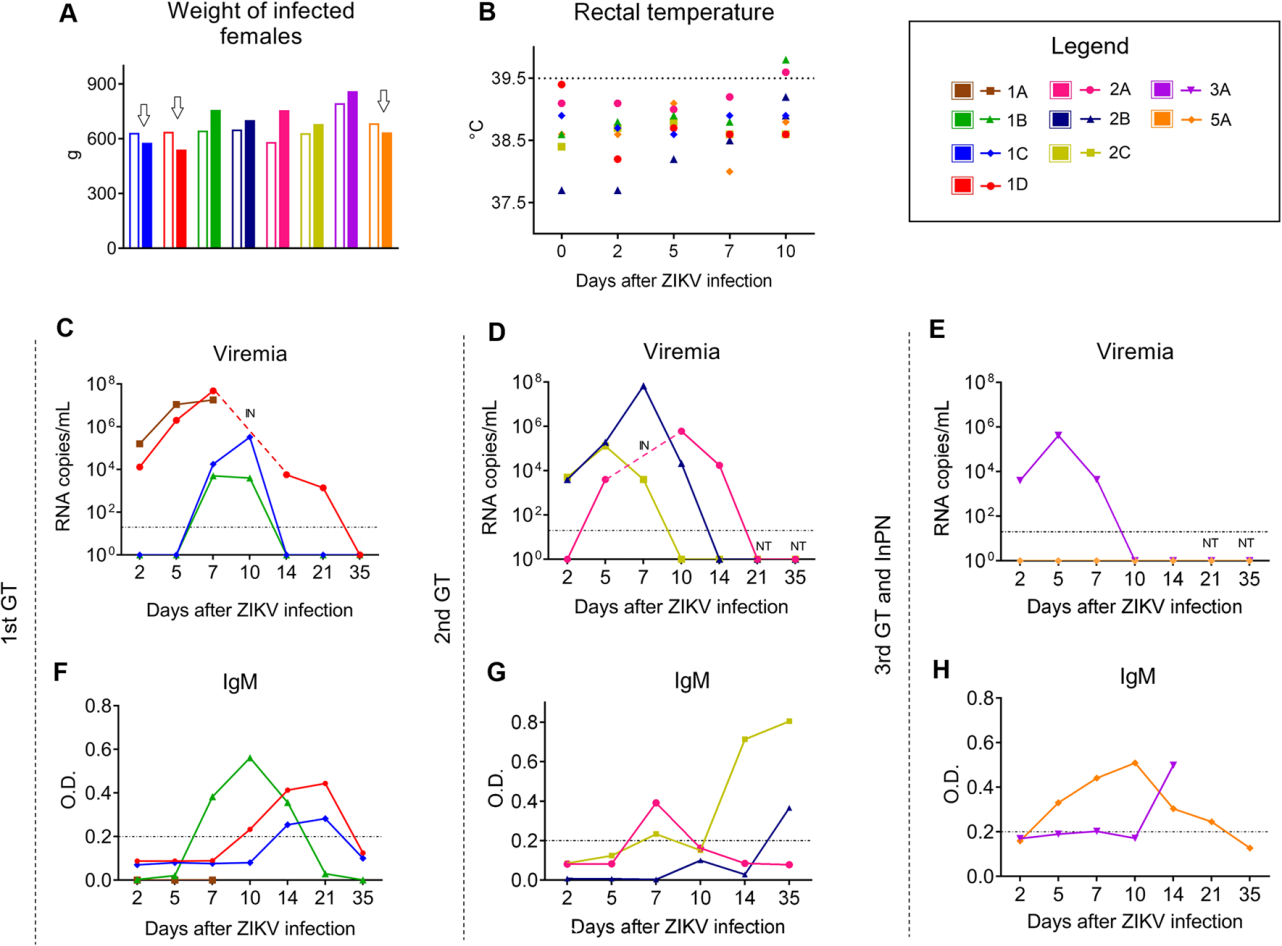
Experimental ZIKV infection in pregnant squirrel monkeys. Clinical ZIKV RNA load in blood and specific antibody profiles in the different infected animal groups. (**A**) Body weight of the females infected with ZIKV before (D0) (unfilled bar) and after infection (D35) (filled bar). Body weight reductions were registered (white arrows) in 1C, 1D and 5A. (**B**) Body temperature of infected pregnant females with ZIKV in the three-thirds of pregnancy and that of the infected nonpregnant monkey. The dotted line indicates rectal temperature (39.5 °C) to indicate the progression of the fever^61^. (**C**–**E**) Detection of viral RNA in blood in the 1st GT, 2nd GT, 3rd GT and InNP groups. (IN: Inconclusive and NT: Not tested). (**F**–**H**) Serologic results of anti-ZIKV IgM antibody detection by ELISA in animals in the 1st GT, 2nd GT, 3rd GT, and InNP groups. The dotted lines represent the limits of detection.

Three infected animals, two from the 1st GT group (1C and 1D) and one from the InNP group (5A), showed mild to moderate loss of appetite resulting in weight loss at approximately 35 days post infection (dpi) (Fig. 2A). At 7 dpi, the 1C animal’s eyelids were swollen, and 1B showed skin suffusions of her right lower limb and capillary fragility at 93 dpi, which persisted for five days. Regarding body temperature, two animals had fever (1B [39.8 °C)] and 2C [39.6 °C]) at 10 dpi (Fig. 2B).

There was a decrease in lymphocytes at 5 dpi and 7 dpi compared to the initial conditions (D0) of the infected animals in the 1st GT (1B, 1C and 1D); however, the hematological and biochemical variable values observed were within reference parameters for the species. The other *S. collinsi* females did not present clinical and hematological changes related to ZIKV infection.

### ZIKV RNA load in blood and specific antibody profile

To check viremia, we performed RT-qPCR^27^ to detect the ZIKV genome in blood or serum samples. Viral RNA was detected in specimens collected from all infected pregnant animals from all GT groups (Fig. 2C–E). Female 1A, which had a miscarriage, presented the highest viral RNA titer observed among the females of the 1st GT group. Interestingly, for 1D, a longer persistence of viremia was observed from 5 to 21 dpi, which was also the highest RNA titer. Animal 1B presented lower and shorter viremia (7 dpi to 10 dpi), and female 1C presented viremia for only two days (Fig. 2C). In the 2nd GT group, the ZIKV genome was detected between 2 and 10 dpi in all animals except for animal 2A, which showed an apparent delay, and RNA was detected at 5 to 14 dpi (Fig. 2D). In the 3A animal (3rd GT), viremia was detected from 2 to 7 dpi, and in the 5A female (InNP), ZIKV was not detected during the experimental days (Fig. 2E). The humoral response was characterized by anti-ZIKV IgM detection in all infected groups (Fig. 2F–H). Interestingly, the 1D and 1C animals showed late IgM detection (at 10 and 14 dpi, respectively), and 1B, which showed low viremia, also presented the highest IgM titers. Up to 7 dpi, anti-ZIKV IgM was not detected in female 1A (Fig. 2F). In the animals infected in the 2nd GT, the highest IgM peak was detected at 35 dpi in the 2C female (Fig. 2G). In the 3A female, antibody detection was performed at 14 dpi, and although the ZIKV genome was not detected in animal 5A, anti-ZIKV IgM was detected from 5 to 21 dpi (Fig. 2H).

### Detection of miscarriage, evaluation of fetal growth, and ZIKV-induced clinical and central nervous system (CNS) damage

In the 1st GT group, 75% of pregnant females had fetal injury detected during US and CT evaluations, including a miscarriage in animal 1A and neurological disorders in the other two fetuses (N-1C and N-1D). In the other groups of pregnant females (2nd GT, 3rd GT and NInP), the fetuses developed without any disorders.

**Figure 3:**
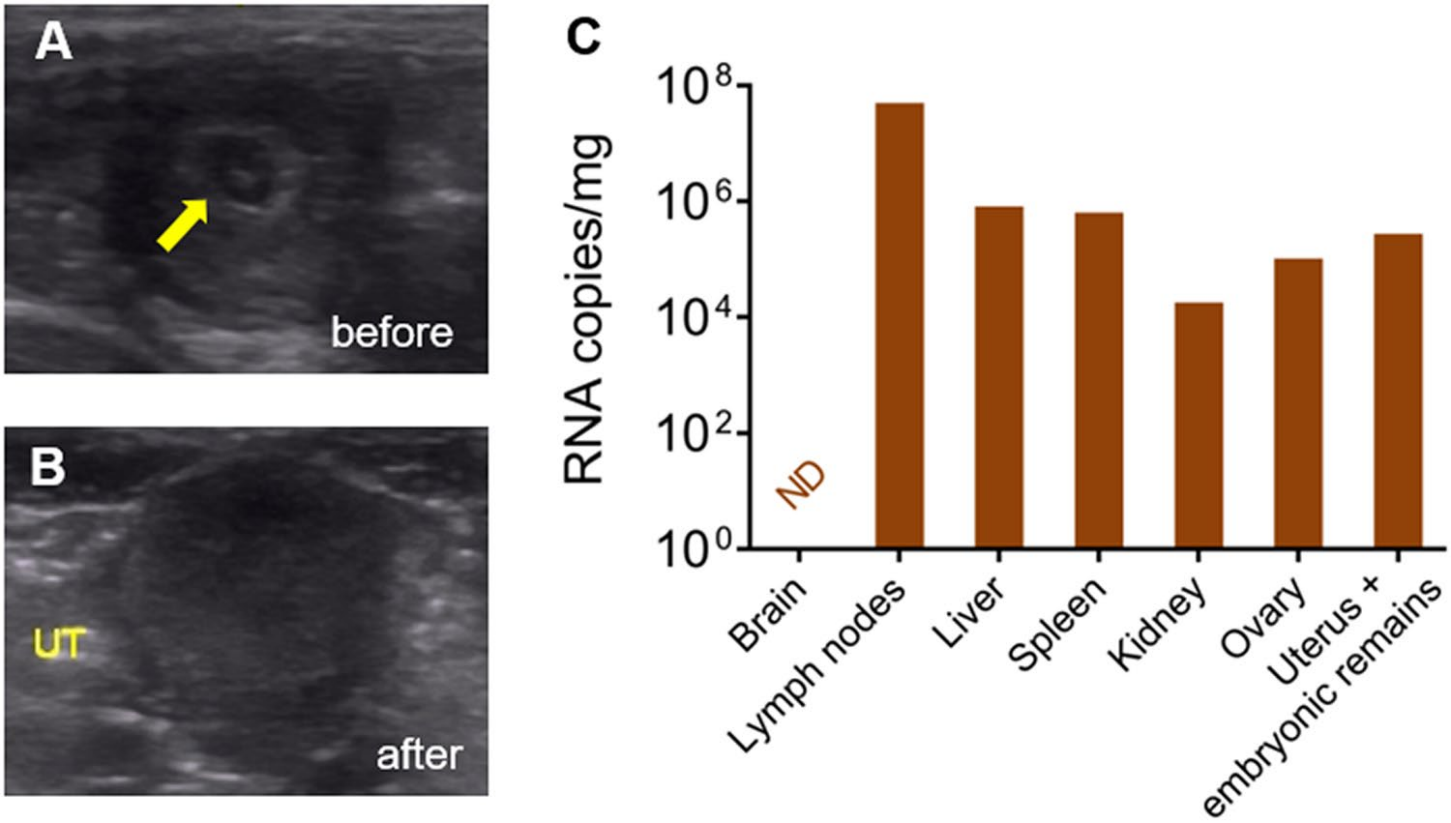
Experimental ZIKV infection in pregnant squirrel monkeys. Miscarriage of the squirrel monkey mother (1A) infected in the 1st GT (34 Gd). (**A**) Ultrasound image at 34 Gd, showing the uterus with a gestational sac and embryo (yellow arrow). (**B**) Ultrasound image at 41 Gd and 7 dpi, showing the uterus with no gestational structures (UT: uterus). (**C**) Detection of viral RNA in tissues from the miscarriage. (ND: Not detected).

The miscarriage in animal 1A was detected by US at 7 dpi relative to 41 Gd. The absence of the gestational sac and embryo was observed by comparing the US data from 34 Gd (1 dpi) (Fig. 3A,B). After euthanasia, performed on the same day as the fetal loss, the organs were harvested, and the ZIKV genome was detected in the lymph nodes, liver, spleen, kidney, ovary and uterus, including in the embryonic remains (Fig. 3C).

For the other animals, US was performed weekly for 23 weeks until delivery. No significant placental abnormalities were detected in any pregnant group. Regarding the fetal parameters, no significant differences were observed in the biparietal diameter (BPD) (H = 2.77; ρ = 0.429), occipital-frontal diameter (OFD) (H = 5.95; ρ = 0.114), cranial circumference (CC) (H = 4.85; ρ = 0.183), or femur length (FL) (H = 2.01; ρ = 0.570) compared to the NInP group.

**Figure 4:**
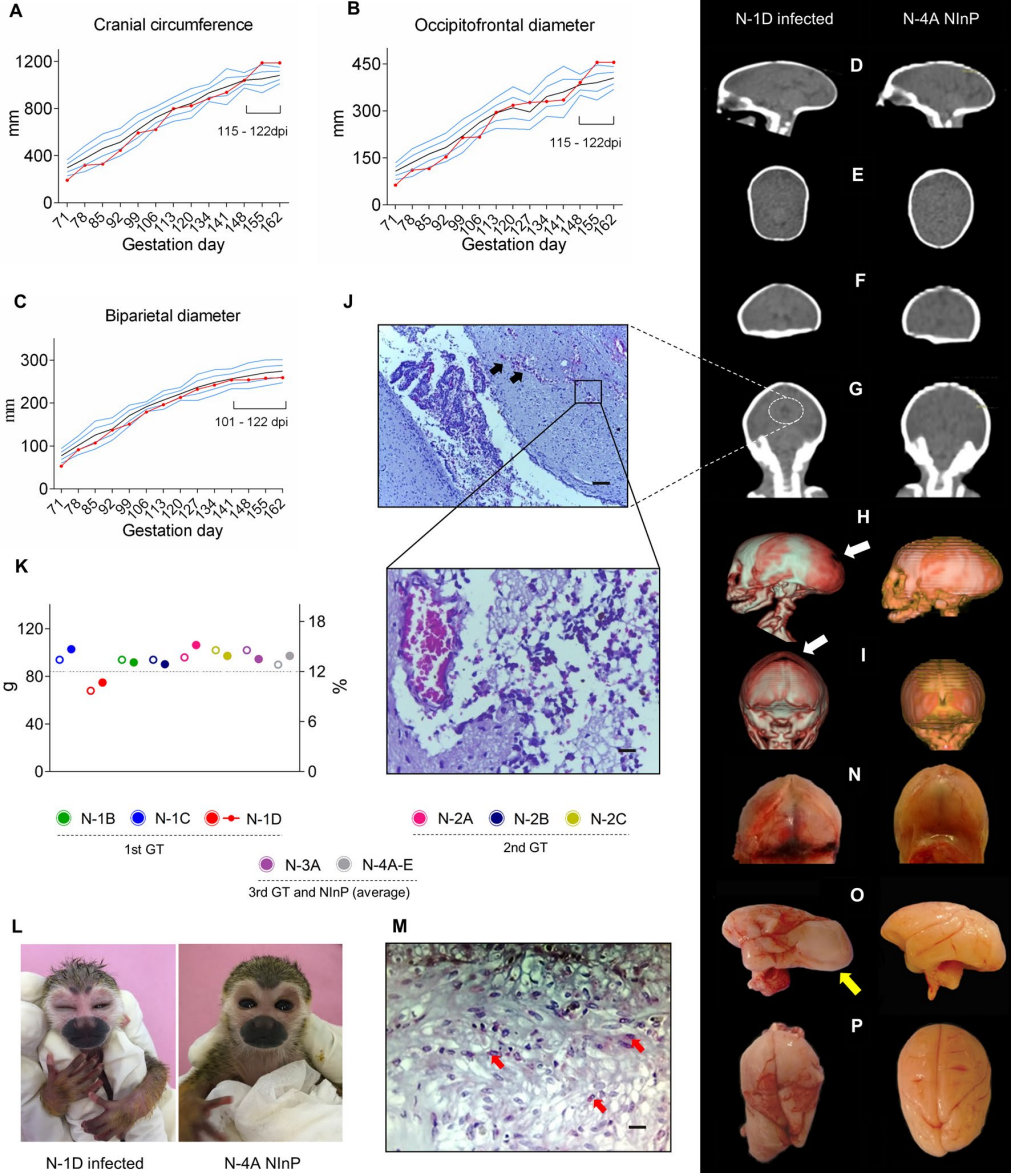
Experimental ZIKV infection in pregnant squirrel monkeys. Fetal growth and neuropathology following vertical ZIKV infection of newborn N-1D whose squirrel monkey mother (1D) was infected in the 1st GT (41 Gd) and comparison to a newborn (N-4A) in the NInP group. (**A**,**C**) Biometric measurements of fetuses at 162 Gd: cranial circumference (**A**), occipitofrontal diameter (**B**) and biparietal diameter (**C**). Average NInP (black line), 1, 2, -1 and -2 standard deviation—SD (blue line) and N-1D animal (red line). (**D**) CT of the skull in the sagittal section showing prominence of the occipital region. (**E**,**F**) Axial and coronal sections of the brain showing bone disproportion. (**G**) Coronal section showing mild right ventriculomegaly (incomplete circle). (**H**,**I**) 3D reconstruction of a brain CT showing posterior fontanelle closure (white arrows). (**J**) Mild ventriculomegaly on the right side, observed hemorrhage (black arrows) (100 × magnification scale bar, 25 µm) and perivascular edema and vasocongestion (box, magnification 400 × scale bar, 50 µm). (**K**) Low weight of N-1D on day 1 of life and proportion to mother's weight. Weight (g) of newborns (open circle). Proportion (%) of newborn in relation to mother's weight (closed circle). Minimum percentage of the newborn's weight in relation to the mother's size (black line)^45^. (**L**) Comparison of infected and uninfected newborns on the 1st day of life. (**M**) IHC markings of ZIKV-positive antigen/antibody reactions in the placenta (red arrows). 400 × magnification; scale bar, 50 µm. (**N**) Gross image of the skull cap with extensive subarachnoid hemorrhage in the posterior fontanelle region. (**O**) Gross brain image showing the incidence in the right occipital region (yellow arrow) and malformation of the right superior temporal sulcus. (**P**) Upper view of the brain showing asymmetry between the two cerebral hemispheres, with an enlargement of the right occipital hemisphere; lissencephaly in the right and left frontal, temporal, parietal and occipital lobes; and hemorrhage in the parietal lobes.

Only the N-1D fetus (1st GT) showed significant fetal growth changes based on its timeline, especially from 115 days into pregnancy. The CC and OFD biometric measurements were more than two standard deviations (SDs) high compared to the average of the NInP group (Fig. 4A,B). This fact was reflected by an increase of 14.5% above the average for the NInP group during the same gestational period. Consequently, the occipital bone was prominent, and a deceleration of BPD was observed in the last two weeks of gestation (Fig. 4C), characterized by craniofacial malformations (Fig. 4D–G). However, this finding did not fulfill the microcephaly criteria (< 2 SDs). During the gestation, the FL was included into the SD range compared to the NInP group, which suggested an absence of symmetrical growth restriction in the neonates.

Cranial CT was performed after the birth of theN-1D offspring. In the sagittal section, the occipital region increased by 4.7% compared to the NInP group (animal N-4A) as well as in the coronal section, where the skull showed bone disproportion with a decrease of 19.3% (Fig. 4D–F). Furthermore, after the cerebral ventricle evaluation, an increase in the right lateral ventricle was observed, measuring 1.43 mm, which was larger than the left ventricle (1.09 mm), characterized as mild ventriculomegaly (Fig. 4G), and in the 3D CT reconstruction image, posterior fontanelle closure was absent (Fig. 4H–I). The histopathological analysis confirmed the ventricular increase, as well as edema, parenchymal hemorrhage and inflammatory cell infiltration (Fig. 4J).

The newborn N-1D weight at birth (68 g) was 24% lower than the control animals’ average body weight (89.6 g). Furthermore, it also had the lowest offspring/mother weight coefficient (10.7) (Fig. 4K). Clinically, the newborn N-1D was lethargic and apathetic towards other newborns (Fig. 4L), leading the mother to reject it on the first day. The newborn subsequently died naturally at 5 days after birth. The other neonates did not present apparent clinical changes and were euthanized at day 5 after birth, and their organs were harvested for histopathological evaluation and comparison.

During the mother’s necropsy (1D), we collected placental remains, and ZIKV was detected by RT-qPCR at high titers (5.9 × 10^7^ RNA copies/mg) as were antigens by immunohistochemistry (IHC) (Fig. 4M). In the neonate’s necropsy (N-1D), a hemorrhagic brain with multifocal areas of subarachnoid hemorrhage was observed (Fig. 4N), along with alterations in the giral pattern and the absence of parenchymal extension grooves and groove deformity, which well evidenced in the superior temporal sulcus (Fig. 4O). Changes such as lissencephaly were also observed, with a practically smooth parenchymal surface seen diffusely between the lobes (Fig. 4P). It is important to note that our imaging findings matched the necropsy data and ventriculomegalies and confirmed craniofacial malformations.

**Figure 5:**
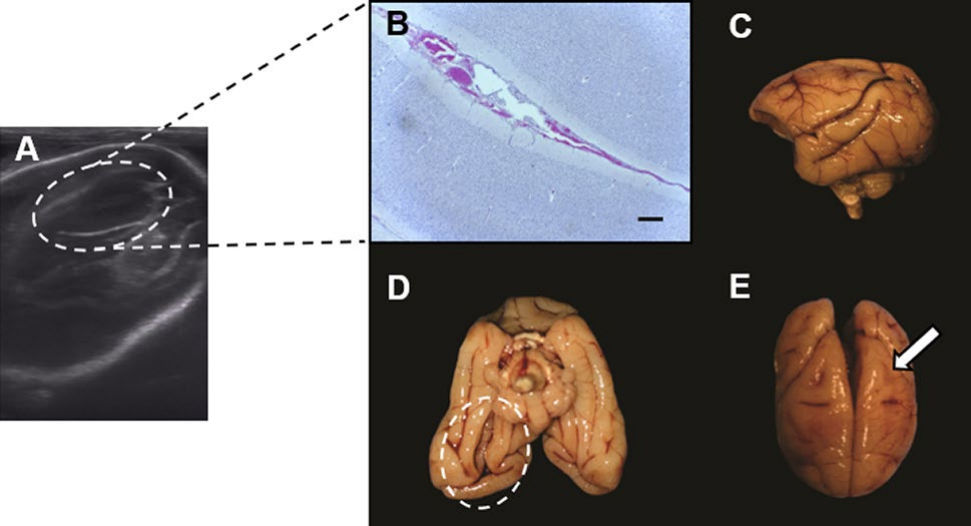
Experimental ZIKV infection in pregnant squirrel monkeys: fetal growth and neuropathology of the newborn vertical ZIKV infection (N-1C) after intradermal infection of its mother (1C) during the 1st GT. (**A**) Ultrasound image showing the area with right lateral ventriculomegaly (incomplete circle). (**B**) Histopathological image showing right ventricular edema and ventriculomegaly (100 × magnification scale bar, 25 µm). (**C**) Gross image of the right brain hemisphere showing marked vessel congestion. (**D**) Crude image of the brain showing distal rotation of the brain in the occipital region and right hemisphere (incomplete circle). (**E**) Gross brain image showing groove deformity in the left hemisphere parietal region (white arrow).

In addition, the newborn N-1C brain showed an increase in the right lateral ventricle (mild ventriculomegaly), observed by US at the end of the 3rd GT (134 Gd) (Fig. 5A); however, the cranial biometric measurements were within the SD for the species. This neonate did not show any behavioral changes. However, during the necropsy analysis, a hemorrhagic brain with groove deformity was observed, which was well evidenced in the sulcus parietal region. The right ventricle was also enlarged and had edema that also appeared in the histopathological section analysis (Fig. 5B–E).

## Discussion

Squirrel monkeys are historically known to be an excellent model for the study of encephalitis^21,22^ and show experimental susceptibility to arbovirus infection^28^. In our study, the adult and newborn monkeys mimicked all the characteristics of ZIKV infection, including those observed in humans. A wide range of clinical manifestations was observed in animals with asymptomatic infection and those presenting mild to severe symptoms that resulted in miscarriages, stillbirths and neurological disorders similar to CZS^8,29,30^. It has also been hypothesized that neotropical NHPs could serve as good hosts and amplifiers of ZIKV in an eventual sylvatic cycle, and the experimental susceptibility of the NHP species to ZIKV infection has proven this assumption^21–23^.

Rodents, including laboratory mice, are not natural hosts for ZIKV infection, and accordingly, their immune responses, through interferon (IFN), can inhibit the replication of ZIKV. As a result, murine models useful for ZIKV infection require IFN manipulation, and therefore, they have inherently limited potential for the study of both translational relevance and congenital pathogenesis mechanisms^17,31–33^. Nonetheless, vertical transmission in mouse models of ZIKV infection has been established, as have pathophysiology studies. However, the maternal–fetal interface, placental structure and course and complexity of fetal brain development of mice are quite different from those of humans^20,32,34,35^.

On the other hand, NHPs are ideal models for studying ZIKV pregnancy infection because, in general, they mimic human pregnancy in several important ways, including placentation and neurodevelopmental time^36,37^. Moreover, the squirrel monkey is able to reproduce in well-defined periods throughout the year with short pregnancies, becoming a viable and efficient pregnancy model for ZIKV infection^25,26^.

ZIKV infection with 10^5^ PFU by the i.d. route was efficient in inducing viremia, the humoral immune response and brain lesions in squirrel monkeys, as has been observed in other neotropical or Old World NHPs^15,37–39^. However, in some NHP models, CZS could not be induced by vertical transmission using i.d. or subcutaneously, even when inoculating high viral infectious doses^23,24^. Thus, the aggressiveness of ZIKV may not only be related to the infectious dose but also be linked to the susceptibility of the species and a combination of several organic factors that induce several different neurological alterations^36^.

In the present study, low fever was observed during the viremic period in 28.5% of the infected pregnant squirrel monkeys, corroborating previous studies with rhesus monkeys^40^. Low fever is one of the ZIKV infection symptoms commonly observed in humans^9^, occurring in approximately 27% of pregnant woman^10^. In addition, weight loss was curiously observed in two females at the 1st GT (1C and 1D). This fact has also been reported in ZIKV-infected pregnant knockout mice^35^.

The longer viremia observed in the pregnant squirrel monkeys (1D female) infected in the 1st GT has been described in a previous study^24^. It is noteworthy that this animal presented the highest viral RNA titer. This apparent persistent maternal viremia may be the result of viral replication in the placental and fetal tissues, both acting as a virus reservoir and both possibly being associated with miscarriages, stillbirths or neurological disorders in newborns as described elsewhere^41^. The N-1D neonate also showed a higher viral RNA titer and antigen detection by IHC in both the placenta and brain^42^.

In our study, 75% of the females in the 1st GT group had maternal–fetal alterations, including one case of miscarriage and two fetuses (N-1C and N-1D) with neurological disorders detected during embryogenesis by US and CT. Miscarriages were also reported in other NHP models and in humans in early pregnancy^37,38,43,44^. Congenital neurological abnormalities in children whose mothers became infected during pregnancy were identified in 55% of infections occurring in the first trimester, with microcephaly occurring in 3% and fetal death in 7%^10^.

In both humans and NHP models, the first trimester, in which embryogenesis occurs, is the critical period with the highest susceptibility to ZIKV infection^37^ because the trophoblast protection barrier in the placenta has not been established yet^45^. Accordingly, new studies clarifying whether virus-induced pregnancy complications are associated with placental vulnerability at a specific time point in the gestational stage or if they are indirectly affected by the severity of maternal disease and their immune response to ZIKV are still needed^45^. Thus, it is relevant to highlight that mothers 1C and 1D, who showed more clinical alterations, higher viremia peaks, and delayed humoral immune responses, had fetuses with numerous brain abnormalities, such as those observed in CZS in human neonates. On the other hand, animal 1B, which had an early (7 dpi) and higher IgM level (peak at 10 dpi) (Fig. 2F), as well as lower viremia (Fig. 2C), delivered offspring (N-1B) without any apparent clinical or neurologic damage detectable by US or macroscopically during necropsy. The N-1D newborn had a low birth weight compared to the weight of the healthy *Saimiri* newborns, also evidenced by the low percentage of weight in relation to the size of the mother^26,46^. These findings are similar to those observed in children with CZS^47^.

The 1D mother abandoned her newborn after birth. This behavior (maternal rejection) is common among NHPs when the offspring have a problem that precludes their survival^48^. In fact, newborn N-1D died 5 days after birth. Although fatal cases are associated with human newborns with microcephaly^6^, this animal had severe neurological alterations and developmental abnormalities compatible with CZS, such as craniofacial malformations^49^. In fact, newborn N-1D showed craniofacial disproportion resulting from an increase in OFD and CC, in addition to a deceleration in BPD growth and the absence of Fontanelle closure.

The frequency of craniofacial disproportion in 48 ZIKV-infected Brazilian babies was 95.8%, that of biparietal depression was 83.3%, and that of prominence in the occipital region was 75%^50^. The cranial collapse is probably due to ventriculomegaly, as suggested elsewhere^51^. This finding was also detected in newborns N-1C and N-1D, both confirmed by histopathological analysis. Other brain damage characteristics of CZS were found in the autopsy, such as brain groove malformations and hemorrhages, similar to those observed in babies born from ZIKV-infected mothers^14^. In addition, lissencephaly was also present in 100% of newborns with microcephaly from mothers infected with ZIKV in early pregnancy^12^, a period when neuronal migration occurs and when there is displacement of neuronal cells from the germinal matrix to the cerebral cortex^52^.

In summary, the neotropical NHP species *S. collinsi* was able to reproduce most of the clinical findings described in ZIKV infection in infants, including CZS, reported during the 2015–2016 Brazilian outbreak. In this study, it was evident that improved prenatal monitoring can suggest major changes in the development of CZS. The amount and persistence of viremia titers, as well as variations in the immune response, were related to the severity of the changes found in the fetuses. In conclusion, squirrel monkeys proved to be an excellent model to study ZIKV infection and CZS and are an ideal model to test vaccine candidates and antiviral drugs.

## Materials and methods

### Animal selection and management conditions

The NHPs used in this study were born in captivity and kept in a breeding shed at the National Primate Center (CENP) at the Evandro Chagas Institute (IEC), Ananindeua, Para State, Brazil. Before the experimental ZIKV infection, all *S. collinsi* females were screened for optimal health conditions, as well as to assess a potential previous exposure to arboviruses (especially flaviviruses) by HI test. The virus panel used was composed of 19 arboviruses that usually circulate in Brazil (Supplementary Table S1). NHPs with antibody titers greater than or equal to 1:20 were not selected for the experimental infection.

During the reproductive period of the species *S. collinsi* (June to November), two males and five females were kept in collective cages (4.75 m × 2.15 m × 1.40 m) to mate. After confirmation of pregnancy by US, the pregnant females were housed in individual stainless-steel cages with a retractable bottom (80 cm × 90 cm × 80 cm) in an isolated room at a P-2 facility and subjected to natural photoperiods. They were fed a balanced daily diet based on fresh fruit and a pelleted diet designed for neotropical NHPs (MEGAZOO P18, Protein 18%, Fiber Max. 6.5%, Betim—MG) and water ad libitum.

### Experimental infection

For the experimental infection, we used fifteen pregnant females from the *S. collinsi* species*,* where nine animals were infected with ZIKV (BE H815744 strain, GenBank KU365780)^5^. This strain was isolated from a patient who had classic symptoms and lived in Paraiba State during the Brazilian outbreak (2016). After three passages in VERO cells for virus amplification, an inoculum was prepared at a dose of 1.0 × 10^5^ PFU/mL in 0.5 mL. The inoculation was performed by i.d. The infected pregnant females were distributed into three groups based on three gestation periods (the 1st GT, 2nd GT and 3rd GT groups). Additionally, six uninfected and pregnant animals served as the negative control (NInP group), while one infected nonpregnant female was considered the positive control (InNP group). In the NInP group, two animals in each GT received inoculation with a VERO cell suspension as a placebo by i.d. (Fig. 1A,B). Throughout the manuscript, to assist in distinguishing neonate animals from their mothers, all neonates are indicated by codes preceded by the letter "N".

All animals were observed twice per day by a trained team able to recognize pain signs, access appetite, stool quality and activity level. Blood samples were collected for hemograms, biochemistry markers and virologic/serologic tests. Fetal development was monitored by US weekly. Five days after the birth of the offspring, the infected females and neonates, as well as the infected nonpregnant females, were euthanized by anesthetics overdose. The other noninfected females and neonates were returned to their home shed (Fig. 1A,B).

### Obstetric evaluation and clinical parameters

Blood samples were collected from all infected and control animals (NInP) before infection (D0) and at 2, 5, 7, 10, 14, 21, and 35 dpi for blood count and biochemistry analysis, viremia load detection by RT-qPCR, and IgM detection by ELISA. All animals were carefully examined for the presence or absence of fever, particularly on blood collection days, by measuring the rectal temperature and examining for rash, conjunctivitis and other signs, and animals were also weighed.

Blood for the hemogram, including leukocyte differential counts, was collected in EDTA tubes and analyzed in an automated hematology analyzer Cell-Dyn Ruby Hematology System (Abbott Diagnostics, Illinois, USA). The analysis of biochemistry markers was performed in an ABX Pentra 60 (Horiba ABX SAS, French). The alanine aminotransferase (ALT), aspartate aminotransferase (AST), gamma glutamyl transferase (GGT), total protein, albumin, alkaline phosphatase, urea, and creatinine levels were also measured.

### RT-qPCR for ZIKV genome detection

The blood and tissues were mixed with PBS pH 7.4 by a Tissuelyser (QIAGEN, Carlsbad, USA) and submitted for viral RNA extraction using a TRIzol Plus RNA Purification kit (Thermo Fisher Scientific, Carlsbad, USA). RT-qPCR was performed as described by Lanciotti et al.^27^ using the QuantiTect Probe PCR Kit (QIAGEN) and the 7500 Fast Real-time PCR System (Thermo Fisher Scientific). A standard curve with a synthetic amplificon target included in the PUC plasmid was used for RNA quantification following methods described by Nunes et al.^53^. The RNase P gene was used as an endogenous internal control^54^. The assay’s limit of detection was 20 RNA copies/mL^27^.

### ELISA to IgM detection

Enzyme-linked immunosorbent assays (ELISAs) were performed to detect anti-ZIKV IgM antibodies in the sera collected from the NHPs as published elsewhere^55–57^ using anti-monkey IgM (KPL, Milford, USA) and anti-flavivirus antibodies conjugated with peroxidase (6B6c-1). The optical density (O.D.) was determined in a spectrophotometer (BioTek, Winooski, USA) using a 450 nm filter, and the cutoff was determined at 0.2.

### Ultrasonography and computed tomography

All pregnant NHP females were submitted weekly to US using the ultrasound machine GE Logic (GE Medical Systems, China) in B mode with the L8-18 MHz linea multifrequency transducer to measure the parameters of the mean gestational sac diameter (MGSD), biparietal diameter (BPD), occipital frontal diameter (OFD), cranial circumference (CC) and femur length (FL). Each parameter was measured three times using an arithmetic mean for the calculation.

The conception date was calculated from the observation intervals of the ultrasound examinations and the average gestation of *S. collinsi*, 162 ± 10 days^26^. After birth, the neonates were weighed and exhaustively evaluated to determine their general health, the condition of their mucous membranes, skin turgor, hearing, vision, suction and vocalization.

The neonates had their skulls examined by CT using BrightSpeed tomography (GE Healthcare, Waukesha, USA) with 64 channels at 120 kV and 250 mAs, with a collimation minimum of 1 mm, reconstruction thickness of 1 mm, image capture field (ICF) equal to 7 cm, and a STND/I filter. For this procedure, the animals were sedated with isoflurane by inhalation anesthesia.

### Histological analysis

CNS anatomical regions were evaluated by optical microscopy (Zeiss, Oberkochen, Germany) at 10X and 40X magnifications by a pathologist and a veterinarian, and histopathological examination was performed on 5-μm sections of paraffinized tissue slides cut and stained with hematoxylin–eosin (HE).

### Immunohistochemistry

The alkaline streptavidin phosphatase method was adapted to detect the viral antigen using an anti-ZIKV polyclonal antibody produced in mice at the IEC. The peroxidase method was based on previous studies^32,58,59^ and used for tissue immunostaining with a specific monoclonal antibody. Slides were examined under a light microscope.

### Statistical analysis

The data and parameters of descriptive statistics, such as the mean and SD, and reference range for the variables of both groups, were determined and stored in spreadsheets, and the statistical analysis was performed using the GraphPad Prism 6.0 program (GraphPad Software, San Diego, USA). For univariate analysis, the frequencies and measures of the central and dispersion parameters were obtained. The Kruskal–Wallis test was used to evaluate the differences between the parameters of BPD, OFD, CC and FL. The level of significance adopted for all the tests was as follows: not statistically significant *p* > 0.05; significant *p* < 0.05; very significant *p* < 0.005; and extremely significant *p* < 0.0005.

### Ethical and legal aspects

All animal experiments were carried out in accordance with the relevant guidelines and regulations. This study was previously approved by the Animal Research Ethics Committee (CEUA) from IEC (CEUA/IEC—n° 010/2016) as well as by Chico Mendes Institute of Biodiversity of the Brazilian Environment Ministry (SISBIO/ICMBio—no. 53391-1) and is in accordance with the provisions of Law No. 11.794 of October 8, 2008, with the provisions of Decree No. 6.899 of July 15, 2009, and with the rules issued by the National Control Council of Animal Experimentation (CONCEA). In addition, the entire experiment was carried out in accordance with adherence to the ARRIVE guidelines.

The euthanasia protocol for NHP mothers and neonates was performed with prior sedation of Ketamine Hydrochloride and Xylazine Hydrochloride intramuscularly, followed by anesthetics overdose with Fentanyl and Ketamine Hydrochloride, intravenously, as by the CONCEA recommendations of the Ministry of Science, Technology and Innovation-MCTIC^60^.

## Supplementary Information

Supplementary Table.
