# Supplementary material for: Prenatal disorders and congenital Zika syndrome in squirrel monkeys
Source: Sci Rep. 2021 Jan 29;11:2698. doi: 10.1038/s41598-021-82028-3 (PMC7846595; doi:10.1038/s41598-021-82028-3)
Supplement: Supplementary file 1 — Supplementary Table. [file 41598_2021_82028_MOESM1_ESM.pdf]

Prenatal disorders and congenital Zika syndrome in squirrel monkeys

Aline Amaral Imbeloni[1,2†], Bianca Nascimento de Alcantara[2], Leandro Nassar Coutinho[3], Sarah Raphaella Rocha de Azevedo Scalercio[1], Liliane Almeida Carneiro[1], Karol Guimarães Oliveira[1], Arnaldo Jorge Martins Filho[4], Darlene de Brito Simith[5], Wellington Bandeira da Silva[1], Bruno Tardelli Diniz Nunes[5], Livia Medeiros Neves Casseb[5], Jannifer Oliveira Chiang[5], Carlos Alberto Marques de Carvalho[5], Mariana Borges Machado[6], Juarez Antônio Simões Quaresma[4,7], Daniele Barbosa de Almeida Medeiros[2,5†*], Pedro Fernando da Costa Vasconcelos [2,5,7†*].

[1]National Primate Center, Evandro Chagas Institute; Rodovia BR-316, km-07, Ananindeua, Para, 67030-000, Brazil.

[2]Evandro Chagas Institute, Post-Graduate Program in Virology, Rodovia BR-316, km-07, Ananindeua, Para, 67030-000, Brazil.

[3]Rural Federal University of Amazonia, Trancredo Neves, 2501, Belem, Para, 66077-830, Brazil.

[4]Evandro Chagas Institute, Department of Pathology, Rodovia BR-316, km-07, Ananindeua, Para, 67030-000, Brazil.

[5]Evandro Chagas Institute, Department of Arbovirology and Hemorrhagic Fever, Rodovia BR-316, km-07, Ananindeua, Para, 67030-000, Brazil.

[6]University Center of Para, Governador Jose Malcher Avenue, 485, Belem, Para, 66035-065, Brazil.

[7]University of Pará State, Tv. Perebebuí-Marco, 2623, Belém, Para State, 66087-662, Brazil.

† These authors have contributed equally to this study

[*]**Corresponding authors:**

Daniele Barbosa de Almeida Medeiros (danielemedeiros@iec.gov.br); **Phone:**+55(91) 3214-2281 Fax: +55(91) 3214-2279.

Pedro F.C. Vasconcelos (pedro.vasconcelos@uepa.br) **Phone:**+55(91) 3214-2281 Fax:+ (91) 3214-2279.

Supplementary Table S1. Arbovirus antigens used in the hemagglutination inhibition test (HI).

Family	Record	Arbovirus
Togaviridae	AN 7526	Eastern equine encephalitis virus (EEEV)
	AN 70100	Western equine encephalitis virus (WEEV)
	AR 20290	Mayaro virus (MAYV)
	AN 10967	Mucambo virus (MUCV)
Flaviviridae	AR 23379	Saint Louis encephalitis virus (SLEV)
	Quimera	West Nile virus (WNV)
	H 111	Yellow fever virus (YFV)
	H 7445	Ilheus virus (ILHV)
	AN 327600	Cacipacore virus (CPCV)
	AN 4116	Bussuquara virus (BSQV)
	H 34675	Rocio virus (ROCV)
Bunyaviridae	AN 73	Tacaiuma virus (TCMV)
	AR 7272	Maguari virus (MAGV)
	AN 24262	Icoaraci virus (ICOV)
	AN 84785	Utinga virus (UTIV)
	AN 141106	Belem virus (BLMV)
	AN 3994	Caraparu virus (CARV)
	AN 19991	Oropouche virus (OROV)
	H 151	Catu virus (CATUV)

SOURCE: SAARB - IEC/SVS/MS- PA.
